# Photographic Atlas of the Diseases of the Skin

**Published:** 1901-12

**Authors:** 


**Photographic Atlas of the Diseases of the Skin.** By GEORGE HENRY FOX, M.D. London: J. B. Lippincott Company. 1901.

Dr. George Henry Fox's *Photographic Atlas of Diseases of the Skin*, of which the plates are coloured, most accurately portrays the lesions of skin affections. We have received parts i. to xii., in which we consider the coloured photographs are excellent and of a convenient size. Opposite each picture there is a description of the condition, with the different synonyms, and in addition to this there are general considerations of the treatment of skin diseases. Under the general considerations it is stated that, owing to the intimate relationship of the skin to other organs, cutaneous disease is usually of internal origin. To this expression of opinion we think some eminent dermatologists will take exception. At the same time, we agree that for the student of dermatological therapeutics the first thing to learn is the great importance of treating the patient, and not merely the patient's skin. It is well said under this heading that "diagnosis implies something more than merely giving a correct name to a pathological condition." On account of its prophylactic rather than its curative power, we endorse the suggestion of a daily bath. As the author says, the main object of a bath is to refresh and invigorate, not merely to cleanse, and that the cool bath taken every morning is capable of producing the greatest benefit. We are delighted to find that exercise as a dermatological remedy is insisted on, and that it is said to accomplish, in a large class of skin diseases, far more than a whole pharmacopœia. In the treatment of acne of the face, facial massage is said to have recently come to be a favourite mode of improving the complexion as well as the acne. For several years past the author has, in the treatment of acne, almost given up the use of ointments and lotions in his private practice, and, so far as local treatment is concerned, has relied almost entirely upon the benefit derived from the use of the ring curette. An enlightened view is taken in connection with the daily washing of the head or wetting of hair with the morning bath. Some authorities think that it has a tendency to induce premature alopecia. No facts, however, have been offered to substantiate this belief; and the author expresses his firm conviction that frequent washing of the scalp, so far as it has any effect upon the growth of hair, is beneficial rather than injurious. As stated too, brushing of the hair should be thoroughly performed twice a day at least. Hair-brushes with thick and long bristles should be chosen; and stiff or wiry ones, which are liable to irritate the scalp, should be avoided. The condition known as pityriasis circinata, pityriasis rosea, lichen circinatus, or seborrhœa corporis, we have always considered to be purely local in origin. It is, however, stated to be undoubtedly of internal origin. It certainly occurs oftener in patients who sweat freely, but further than this we cannot go. In another edition it would be more satisfactory if the reasons for this opinion were given. Pemphigus is sometimes controlled by arsenic, which is often a remedy of great value, but we agree that it is by no means a specific in this disease.

The majority of cases of alopecia areata are evidently thought not to depend upon a parasite. But the current view among London dermatologists is, that a parasite is responsible for 95 per cent. of the cases. It certainly tends to occur in epidemic form in regiments and schools.

The printing is most clear, and the book a most readable one; and with the very beautiful and accurate plates, it will, we feel sure, be a valuable work for practitioners and students of medicine.

